# Corneal Collagen Cross-Linking

**DOI:** 10.4103/0974-9233.61213

**Published:** 2010

**Authors:** Mirko R. Jankov II, Vesna Jovanovic, Ljubisa Nikolic, Jonathan C. Lake, Georgos Kymionis, Efekan Coskunseven

**Affiliations:** Laser Focus-Centre for Eye Microsurgery; 1 KBC Zvezdara, Belgrade, Serbia; 2 OFTALMED Hospital da Visão de Brasilia, Brasilia, Brazil; 3 3Institute of Vision and Optics, Department of Medicine, University of Crete, Crete, Greece; 4 Dunya Eye Hospital, Istanbul, Turkey

**Keywords:** Corneal Collagen Cross-Linking, Corneal Ectasia, Corneal Ulcer, Intracorneal Ring Segments, Keratoconus, Topography-Guided Photoablation

## Abstract

Corneal collagen cross-linking (CXL) with riboflavin and ultraviolet-A (UVA) is a new technique of corneal tissue strengthening by using riboflavin as a photosensitizer and UVA to increase the formation of intra and interfibrillar covalent bonds by photosensitized oxidation.

Keratocyte apoptosis in the anterior segment of the corneal stroma all the way down to a depth of about 300 microns has been described and a demarcation line between the treated and untreated cornea has been clearly shown. It is important to ensure that the cytotoxic threshold for the endothelium has not been exceeded by strictly respecting the minimal corneal thickness. Confocal microscopy studies show that repopulation of keratocytes is already visible 1 month after the treatment, reaching its pre-operative quantity and quality in terms of functional morphology within 6 months after the treatment. The major indication for the use of CXL is to inhibit the progression of corneal ectasias, such as keratoconus and pellucid marginal degeneration. CXL may also be effective in the treatment and prophylaxis of iatrogenic keratectasia, resulting from excessively aggressive photoablation. This treatment has also been used to treat infectious corneal ulcers with apparent favorable results. Combination with other treatments, such as intracorneal ring segment implantation, limited topography-guided photoablation and conductive keratoplasty have been used with different levels of success.

## INTRODUCTION

Corneal CXL, or C3R, is certainly a topic that has raised a significant interest since its first application more than 10 years ago. In 2009 alone, there were 49 publications related to this topic compared to a mere six publications in 2005.

## CORNEAL COLLAGEN CROSS-LINKING WITH RIBOFLAVIN AND ULTRAVIOLET-A

Corneal CXL with riboflavin and UVA is a new technique of corneal tissue strengthening using riboflavin as a photosensitizer and UVA to increase the formation of intra-and interfibrillar covalent bonds by photosensitized oxidation.^1^

The major indication for the use of CXL is to inhibit the progression of corneal ectasias, such as keratoconus and pellucid marginal degeneration.^1^–^10^ CXL may also be effective in the treatment and prophylaxis of iatrogenic keratectasia, resulting from laser *in situ* keratomileusis.^11^–^12^ This treatment has also been used to treat infectious corneal ulcers with apparently favorable results.^13^–^20^ CXL has also been used in combination with other treatments, such as intracorneal ring segment implantation^21^–^23^ and limited topography-guided photoablation, with some success.^24^–^25^

*In vitro* studies have shown that the cornea absorbs approximately 30% of UVA light while an additional 50% of UVA absorption occurs in the lens.^1^ Corneal UVA absorption can be considerably increased with a photosensitizer such as riboflavin. With an irradiance of 3 mW/cm^2^ of UVA and 0.1% riboflavin, as much as 95% of UVA light will be absorbed within the cornea. This results in a 20-fold reduction of the original irradiance of 3 mW/cm^2^ of UVA (at the corneal surface) down to 0.15 mW/cm^2^ (at the endothelial level), which is well below 0.36 mW/cm^2^, the threshold considered cytotoxic for the endothelium.^26^–^28^

By comparison, the same UVA irradiance at the corneal surface as used in the aforementioned studies can be measured at noon during an average sunny summer day in the tropics (23 of latitude and 800 m above sea level).

Despite the expected reduction of irradiance from the corneal surface toward the deeper layers of corneal stroma, the irradiation levels still exceed the threshold down to a depth of approximately 300 microns. Therefore, keratocyte apoptosis in the anterior stromal layer has been described and a demarcation line between the treated and untreated cornea has been clearly shown in both *in vitro* and *in vivo* studies.^26^–^29^

Using a wavelength of 360–370 nm with an accumulated irradiance of 5.4 J/cm^2^ ensures that the exposure of all structures is below harmful levels.^1^ However, a non-homogenous irradiation field may create localized hot spots of increased radiance with potentially harmful consequences. Therefore, for clinical use, a uniformly emitting irradiance source is required, and must be continuously evaluated. The riboflavin in the cornea itself also serves as a further protective layer, which has been reported to reach more than 400 µm after 30 min of application, penetrating the anterior chamber, where it is visible with the slitlamp as a yellow flare. Therefore, there is an UV absorption coefficient that shields the more posterior structures such as the endothelium, the crystalline lens and the retina.^1^

Confocal microscopy shows the repopulation of keratocytes by 1 month after treatment, reaching their pre-operative quantity and quality in terms of functional morphology within 6 months after treatment.^30^

Kymionis *et al*. described the indirect effect of CXL through the change in corneal thickness during and after the treatment due to a more compact and rigid cornea.^31^ Kymionis *et al*. found a statistically significant decrease (mean, 75 microns) in central corneal thickness at the interval of the epithelial removal (415.7 ± 20.6 microns) and at the end of riboflavin solution instillation (340.7 ± 22.9 µm; *P <* 0.001) and no statistically significant change during irradiation (*P >* 0.05).^31^ Pre-operative and 1-month post-operative endothelial cell count were not statistically different (pre-operative, 2780 ± 197 to 1-month post-operative, 2713 ± 116; *P =* 0.14).

Whether repeat treatment may be necessary due to corneal collagen turnover remains unanswered.

## SURGICAL TECHNIQUE

The treatment procedure should be performed under sterile conditions in an operating theater. The currently accepted treatment protocol includes deepithelialization for efficient penetration of riboflavin due to the incomplete absorption of riboflavin by the epithelium because of tight junctions. This method has been successfully used for the treatment of progressive keratoconus and pellucid marginal degeneration since 1999 and for iatrogenic keratectasia since 2003. Published and peer-reviewed data on the safety and efficacy of these parameters for cross-linking are available from numerous research groups, with long-term results out to 6 years.^1^ In the standard technique, removal of the epithelium is required in order to expose the underlying stroma for a complete absorption of riboflavin.

### Cross-linking with removal of the epithelium

Abrasion of the corneal epithelium out to 7 mm is performed under topical anesthesia. Prior to the treatment itself, ultrasound pachymetry should be performed at the thinnest point of the deepithelialized cornea, to ensure a minimal corneal thickness of 400 µm. Riboflavin solution, 0.1% in 20% dextran (Peshckemed, Huenenberg, Switzerland), is then applied to the cornea every 3 min for 30 min. The saturation of the cornea with riboflavin and its presence in the anterior chamber is monitored closely by slitlamp inspection prior to treatment. Riboflavin saturation ensures the formation of free radicals whereas riboflavin shielding ensures the protection of deeper ocular structures such as the corneal endothelium.

**Figure 1:**
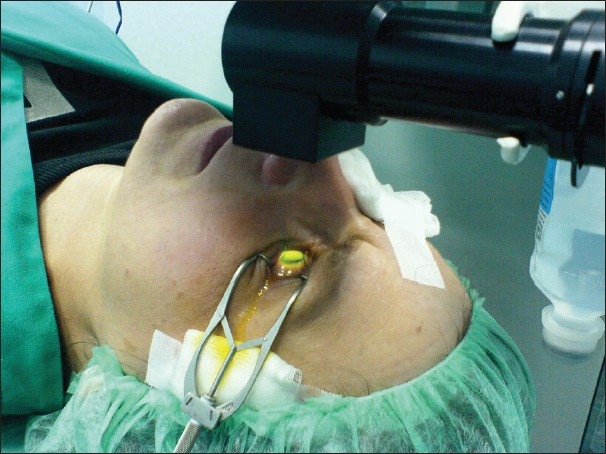
Treatment in progress with the cornea soaked with riboflavin and irradiated by the ultraviolet lamp

UVA irradiation is performed using an optical system (Koehler type illumination) consisting of an array of seven UVA diodes with a potentiometer in series to allow for regulation of voltage (UV-X, Peschkemed, Huenenberg, Switzerland). Prior to treatment, the intended irradiance of 3 mW/cm^2^ surface irradiance (5.4 J/cm^2^ surface dose) is calibrated using a UVA meter (LaserMate-Q; LASER 2000, Wessling, Germany) at a working distance of 6 cm. Irradiance is performed for 30 min using 3 mW/cm^2^, corresponding to a surface dose of 5.4 J/cm^2^. During the procedure, riboflavin solution and topical anesthetic (oxybuprocaine 0.4%) is applied every 2-3 min to saturate the cornea with riboflavin and for corneal hydration [Figure 1].

After the treatment, one drop of topical ofloxacin 0.3% (Exocine, Allergan, Irvine, CA, USA) is instilled and a bandage contact lens is placed until complete reepithelialization. The patients are instructed to instill topical ofloxacin 0.3% four times daily until contact lens removal. In most cases, the contact lens is removed on the third day after treatment. The patient is then instructed to instill topical dexamethasone phosphate 0.1% (Maxidex, Alcon-Couvreur, Belgium) four times daily followed by a tapering schedule over 2 months as follows: Four times daily during the first month, decreasing to two times and then to once a day during the second month.

### CXL without removal of the epithelium

A cross-linking procedure without epithelial removal would likely be less painful than one with the large diameter epithelial removal described above and would be ideal if it efficiently stabilized keratectasia.

Several substances have been used to loosen the tight junctions of the epithelial layer and thus increase the penetration of riboflavin. One is a riboflavin solution containing benzalkonium chloride (BAK), the most commonly used preservative in ophthalmic medications. BAK is also a tensioactive substance, surfactant or an active surface agent that changes the surface tension value, and hence would facilitate the penetration of substances through the epithelium. Currently, there are no peer-reviewed studies that present data on this approach. However, in a comparative *in vitro* study, Samaras *et al*. compared 20% alcohol, partial or complete epithelial removal by analyzing light transmission properties of porcine corneas after CXL and concluded that complete removal of the corneal epithelium appears to be necessary to allow sufficient riboflavin absorption into the stroma to alter the normal light transmission properties of the porcine cornea.^32^ Although partial grid-pattern epithelial removal allows some riboflavin penetration, uptake is limited and irregular, which may affect the efficacy of the cross-linking process.

## APPLICATIONS AND CLINICAL RESULTS

It is important to keep in mind that CXL likely “stops” or “reduces,” rather than reverses, the progression of the keratoconus. Mild regression that occurs may be explained as an effect of the rearrangement of corneal lamellae and the surrounding matrix.^1^ Due to an increased number of cross-linking sites within the collagen molecule after CXL, stiffer fibrils and lamellae are probably generated. This process produces a rearrangement of corneal lamellae and a relocation of the surrounding matrix, which, in turn, results in the reduction of the central corneal curvature.

### CXL in keratoconus

The first *in vivo* controlled clinical study by Wollensak *et al*., which included 23 eyes with moderate or advanced progressive keratoconus, showed that CXL was effective in halting the progression of keratoconus over a period spanning 4 years.^2^ In this study, a mean pre-operative progression of keratometry (max K) by 1.42 D in 52% of the eyes over a 6-month period immediately prior to the treatment was followed by a post-operative decrease in 70% of the eyes.^2^ The statistics also revealed a reduction of the max K by 2.01 D, while the SEQ was reduced by an average of 1.14 D.^2^ In contrast, 22% of the untreated fellow control eyes had a post-operative progression of keratectasia by an average of 1.48 D.^2^

Results from a study by Coscunseven *et al*. confirm Wollensak *et al*.'s findings; the group treated with CXL showed a similar mean decrease in SEQ of 1.03 ± 2.22 D (−5.25 D to + 3.75 D), decrease in cylinder by 1.04±1.44 D (−2.00 D to 4.00 D) and decrease in max K by 1.57 ± 1.14 D (0.00 D to 3.90 D).^6^ In the Coscunseven *et al*. study, the non-treated group showed progression of all corneal parameters under study.^6^

A study by Jankov *et al*. found that progression of keratoconus stopped in all patients who were actively progressing 6 months prior to treatment.^7^ Max K decreased by more than 2 D (from 53.02 ± 8.42 D to 50.88 ± 6.05 D), SEQ decreased from − 3.27 ± 4.08 to − 2.68 ± 3.02 D, while refractive cylinder decreased by < 0.5 D (from − 2.29 ± 1.77 to −1.86 ± 0.92 D).^7^ After treatment, no eyes lost lines of best spectacle-corrected acuity (BSCVA), 12 maintained BSCVA, one gained one line of BSCVA, five gained two lines of BSCVA and one patient gained three lines of BSCVA.^7^

Agrawal found similar results in 37 eyes of Indian subjects 1 year after treatment.^8^ Agrawal reported that 54% of the eyes gained at least one line of BCVA, astigmatism decreased by a mean of 1.2 D in 47% of the eyes, the keratometry value at the apex decreased by a mean of 2.73 D in 66% of the eyes and the maximum K value decreased by a mean of 2.47 D in 54% of the eyes.^8^ In their preliminary results, Wittig-Silva *et al*. found similar results of BSCVA and K readings, with no difference in spherical equivalent and endothelial cell density between treated and control eyes 12 months after CXL.^9^ Vinciguerra *et al*. found that CXL treatment was effective in reducing corneal and total wavefront aberrations 1 year post-operatively.^1^

### CXL in ectasia

Corneal cross-linking has also been used successfully in stopping the advancement of iatrogenic ectasia after excimer laser ablation. In a recently published study, CXL was performed in 10 patients with previously undiagnosed *forme fruste* keratoconus or pellucid marginal corneal degeneration that underwent LASIK for myopic astigmatism and subsequently developed iatrogenic keratectasia.^11^ CXL led to an arrest and/or even a partial reversal of keratectasia over a post-operative follow-up period of 1 year^1^ to 25 months.^11^ The differences were demonstrated with pre-operative and post-operative corneal topography and a reduction of max K.^11^

### Corneal collagen cross-linking in corneal ulcers and edema

The CXL is a promising technique for treating corneal melts or infectious keratitis because cross-linking would strengthen a collagenolytic cornea while UVA irradiation eliminates the infectious agent.^13^ In an experimental study, Schrier *et al*. tested the antibacterial action of riboflavin alone, ultraviolet light alone and the combination of riboflavin and UV light on *Staphylococcus aureus*, methicillin-resistant *S. aureus* (MRSA) and *Pseudomonas aeruginosa*.^14^ Although all plates exposed to riboflavin alone showed no bacterial death and two of five *P. aeruginosa* plates exposed to ultraviolet light alone showed minimal inhibition, all plates exposed to the combination of riboflavin and ultraviolet light showed bacterial death.

CXL of the cornea has been shown to have an anti-edematous effect in the cornea. Wollensak *et al*. presented a case series of three eyes with bullous keratopathy due to pseudophakia, corneal transplant rejection and Fuchs’ endothelial dystrophy that underwent CXL.^15^ After dehydration for 1 day using 40% glucose, the standard CXL technique was used by Wollensak *et al*. for treatment. Corneal thickness was reduced by 90.33 ± 17.04 microns three days after cross-linking and by 93.67 ± 14.22 microns 8 months after CXL. The bullous changes of the epithelium were markedly improved, with the patients reporting no pain or discomfort after CXL.^15^–^17^ Visual acuity was significantly improved in a case without prior stromal scarring. In such cases, CXL is primarily suited for patients with pain symptoms, restricted visual prognosis or to extend the time interval for an upcoming corneal transplantation.

### Other potential applications

Although CXL resulted in a decrease of SEQ, astigmatism and max K, uncorrected visual acuity (UCVA) and BSCVA increased only modestly in the majority of studies of CXL for keratoconus. Other studies with alternative treatment methods for keratoconus, such as implantation of intracorneal rings, have reported more than a two-line increase in BSCVA.^33^–^34^

These observations lead us to the following hypothesis: If the treatment with CXL stops or slows the progression of keratoconus, while other methods can reshape the cornea, a logical solution would be to combine the two treatment methods in order to synergize their effects. In this combined method, a pre-treatment with an alternative method would significantly reshape the cornea by flattening and regularizing corneal shape, which would be followed by CXL to stabilize the cornea. Alternatively, the CXL procedure could be performed first, followed by a reshaping procedure.

### CXL combined with intracorneal rings

Kamburoglu *et al*.^22^ reported a case of post-operative LASIK ectasia that underwent Intacs SK implantation and CXL treatment in both eyes. The pre-operative BSCVA was 20/60 in the right eye and 20/80 in the left eye. The pre-operative spherical equivalent was −14.50 D in the right eye and − 10.50 D in the left eye. Mean keratometry pre-operatively was 56.20 D in the right eye and 50.70 in the left eye. Following bilateral Intacs SK implantation, CXL was performed the following day in the left eye and after 1 month in the right eye. Eight months after combined treatment, BSCVA was 20/25 and 20/25, manifest refractions were − 1.50 × 170 and − 1.25 50 and mean keratometric values were 47.20 and 44.20 D in the right and left eyes, respectively.

In 2007, Chan *et al*. performed a retrospective, non-randomized, comparative case series of 12 eyes of nine patients who had inferior-segment INTACS placement without CXL and 13 eyes of 12 patients who had inferior-segment INTACS placement followed by CXL.^23^ The INTACS with CXL group had a significantly greater reduction in cylinder than the INTACS-only group and there was a significantly greater reduction in max K in the INTACS with CXL group.^23^ Chan *et al*. concluded that the addition of CXL to the INTACS procedure resulted in greater improvements than INTACS insertion alone for keratoconus cases.

We have previously conducted a prospective, comparative study that comprised 48 eyes of 43 patients with progressive keratoconus.^21^ In Group 1, CXL was performed first, followed by intracorneal rings (ICR) implantation.^21^ In group 2, intracorneal rings implantation was performed first, followed by CXL.^21^ The mean interval between the steps was 7 months, while mean follow-up after the second step was 6 months.^21^ In Group 1, the outcomes after the CXL-only treatment were similar results to previous reports for CXL treatments only: An increase in UCVA and BSCVA of approximately one line and half a line respectively, and a decrease in SEQ, mean cylinder and mean keratometry of 1.39 D, 0.44 D and 0.88 D, respectively.^21^ In group 2, where CXL was applied to a cornea with ICR in place, the results show a similar increase in UCVA and BSCVA and a decrease in cylinder, while there was a statistically insignificantly smaller increase in SEQ and insignificantly higher decrease in max K.^21^

Therefore, CXL treatment showed a similar effect when applied over the cornea with ICR already in place as it had on with CXL-only treatments, having a similar modest improvement in all corneal parameters.^21^ However, longer follow-up is warranted to determine whether the biomechanical effect reported in CXL-only treatments also affects the combined treatments.

Compared to the modest reduction in spherical equivalent, cylinder and max K after CXL only, other treatment methods for keratoconus, such as intracorneal rings, show greater improvement of corneal parameters. Miranda *et al*. reported a reduction of spherical equivalent and max K of more than 2.50 D and 6.00 D, respectively, using Ferrara rings.^34^ Miranda's study included 26 eyes diagnosed with keratoconus grades 3 and 4.^34^ The Ferrara rings flattened the central and peripheral cornea, displacing the corneal apex to its physiological position in front of the pupil by reducing the paracentral ectasia commonly present in keratoconic corneas.

Our results in group 2, with implantation of ICR in an intact cornea, expectedly show the same results as in the literature for ICR only: Increase in UVA and BSCVA of about two lines and three lines and a decrease in SEQ, cylinder and mean K of 3.31 D, 2.05 D and 2.94 D, respectively.^21^ In group 1, where ICR were implanted after a previous treatment with CXL, the results show a similar, however slightly smaller increase, in UCVA and mean K, while there was a statistically significantly smaller increase in BSCVA of one line.^21^ There was a statistically significantly smaller decrease of SEQ and cylinder by 2.76 D and 1.32 D, respectively.^21^

Therefore, ICR implantation showed a greater effect when applied over an intact cornea rather than on a cornea already treated with CXL, although showing improvement in all the corneal parameters in either treatment sequence.^21^

Considering the overall effect of joint treatments, group 2 showed a higher overall increase in BSCVA and cylinder (*P < *0.01) as well as a higher but statistically insignificant overall increase in UCVA and decrease in spherical equivalent compared with group 1. These findings suggest that although each of the treatment steps demonstrates the improving effect on the cornea, a stiffer cornea already treated by CXL somewhat inhibits the flattening forces of ICR, thus restricting their effect and decreasing the maximal flattening potential.^21^ In order to achieve the maximal overall effect, ICR should be applied first, allowing the reshaping of the cornea unrestricted, after which the additional CXL treatment should be applied to additionally flatten the cornea and biomechanically stabilize it.

Combination of ICR implantation with ultraviolet/ riboflavin-mediated corneal CXL procedure seems to have a synergic effect for reverting the progressive irregular astigmatism due to keratoconus or iatrogenic ectasia. Implantation of ICR followed by the CXL resulted in greater keratoconus improvements than the CXL procedure followed by ICR implantation.^21^

### Corneal collagen cross-linking combined with limited topoguided photorefractive keratectomy

One of the most promising uses of the CXL procedure is in combination with a modified version of PRK. In a prospective study, Kanellpoulos included a total of 325 eyes with keratoconus.^24^ The first group (*n = *127 eyes) underwent CXL with subsequent topography-guided PRK performed 6 months later (sequential group) and the second group (*n = *198 eyes) underwent CXL and PRK in a combined procedure on the same day (simultaneous group) using the Allegretto (WaveLight, Erlangen, Germany) topography-guided laser platform to normalize the shape of the cornea. Statistically, the simultaneous group performed better (*P < *0.05) in all parameters evaluated, including UCVA and BSCVA, spherical equivalent refraction and keratometry, and less corneal haze.^24^

In a similar prospective study, Kymionis *et al*. reported favorable results of 14 eyes with progressive keratoconus that were treated with customized topography-guided PRK with the Pulzar Z1 (wavelength 213 nm, CustomVis) immediately followed by CXL.^25^

It is important to emphasize that combined treatment of CXL/PRK is a specialized inter vention with the goal of normalizing the cornea as much as possible to increase BSCVA rather than treating the refractive error itself. Therefore, the primary treatment target is cylinder in order to improve the irregular astigmatism and the secondary target is correcting some of the sphere. Most importantly, the eye may not require a corneal transplant.

### Corneal collagen cross-linking combined with conductive keratoplasty

Kymionis *et al*. recently showed that corneal remodeling with conductive keratoplasty in patients with keratoconus seems to have a temporary effect despite the subsequent application of CXL in two patients with keratoconus.^35^ Conductive keratoplasty spots were applied on the flatter side of the cornea followed by CXL. Immediately after conductive keratoplasty, a significant corneal topographic improvement was observed. However, the effect of conductive keratoplasty regressed 3 months post-operatively and remained unchanged until the sixth post-operative month in both patients.

## COMPLICATIONS AND OTHER CONSIDERATIONS

Although CXL is a minimally invasive method, recent reports have indicated possible adverse effects. In a recent retrospective study of 163 eyes of 127 patients with stage 1-3 keratoconus, 8.6% developed a clinically significant haze after 1-year follow-up.^36^ However, the mean pre-operative keratometry (K) value of the apex in the haze group was 71.1 ± 13.2 D compared with 62.1 ± 13.8 diopters (D) in the control group, and the mean corneal thickness was 420.0 ± 33.9 microns in the haze group compared to 478.1 ± 52.4 microns in the control group.^36^ Therefore, advanced keratoconus should be considered at higher risk of haze development after CXL due to low corneal thickness and high corneal curvature.

Mazzotta and colleagues have also presented two cases of post-operative corneal haze among a cohort of 40 eyes of 39 keratoconus patients.^37^ In two cases, stromal haze appeared 2-3 months post-operatively and was resistant to topical steroid treatment. Repeated examination of the pre-operative confocal studies of these patients revealed a reticular pattern of stromal microstriae that may imply advanced keratoconus.^37^ However, even with the haze, BSCVA in these patients was improved.^37^ The authors report confocal findings that possibly reflect keratoconus and they suggest them to be a relative contraindication to performing CXL.^37^

Additional case reports describe diffuse lamellar keratitis^38^ and a reactivation of herpetic keratitis^39^ following CXL. In both cases, prompt diagnosis and treatment resulted in favorable resolution. In another report, Koppen *et al*. reported four cases of keratitis and corneal scarring from a total of 117 eyes treated with CXL where patients experienced delayed (more than 24 h) symptoms and signs of inflammation.^40^ The eyes showed pronounced ciliary redness with cells in the anterior chamber and central keratic precipitates; multiple white infiltrates had developed at the edge and within the area of CXL.^40^ In two eyes, there was a persistent decrease in BSCVA even after rapid initial improvement of symptoms and signs following high-dose topical or subconjunctival corticosteroids.^40^

## CONCLUSIONS

Corneal CXL mediated by riboflavin and UVA appears to be a safe and efficacious procedure in halting the progression of keratoconus and iatrogenic ectasia. CXL reduces the corneal curvature, spherical equivalent refraction and refractive cylinder in eyes with corneal instability and progressive irregular astigmatism due to keratoconus and ectasia. The CXL technique is promising in treating corneal melting conditions or infectious keratitis because cross-linking would strengthen a collagenolytic cornea while UVA irradiation eliminates the infectious agent. Combination of ICR implantation with CXL seems to have a synergistic effect for reverting the progressive irregular astigmatism due to keratoconus or iatrogenic ectasia. A sequential or simultaneous combination of limited topography-guided PRK and CXL, whose goal is normalizing the cornea as much as possible, shows promising results.

## References

[1] Spoerl E, Huhle M, Seiler T (1998). Induction of cross-links in corneal tissue. Exp Eye Res.

[2] Wollensak G, Spoerl E, Seiler T (2003). Riboflavin/Ultraviolet-A-induced collagen crosslinking for the treatment of keratoconus. Am J Ophthalmol.

[3] Kohlhaas M, Spoerl E, Speck A, Schilde T, Sandner D, Pillunat LE (2005). A new treatment of keratectasia after LASIK with riboflavin/UVA light cross-linking. Klin Monatsbl Augenheilkd.

[4] Caporossi A, Baiocchi S, Mazzotta C, Traversi C, Caporossi T (2006). Parasurgical therapy for keratoconus by riboflavin-ultraviolet type A rays induced cross-linking of corneal collagen: Preliminary refractive results in an Italian study. J Refract Surg.

[5] Mazzotta C, Balestrazzi A, Traversi C, Baiocchi S, Caporossi T, Tommasi C (2007). Treatment of progressive keratoconus by riboflavin-UVA-induced cross-linking of corneal collagen: Ultrastructural analysis by heidelberg retinal tomograph II in vivo confocal microscopy in humans. Cornea.

[6] Coskunseven E, Jankov MR, Hafezi F (2009). Comparative study of corneal collagen cross-linking with Riboflavin and UVA irradiation in patients with keratoconus. J Refract Surg.

[7] Jankov MR, Hafezi F, Beko M, Ignjatovic Z, Djurovic B, Markovic V (2008). Ultra B2-promoção de ligaç[otilde]es covalentes do colágeno cornea (corneal cross-linking) no tratamento de ceratocone: Resultados preliminares. Arq Bras Oftalmol.

[8] Agrawal VB (2009). Corneal collagen cross-linking with riboflavin and ultraviolet: A light for keratoconus: Results in Indian eyes. Indian J Ophthalmol.

[9] Wittig-Silva C, Whiting M, Lamoureux E, Lindsav RG, Sulivan IJ, Snibson GR (2008). A randomized controlled trial of corneal collagen cross-linking in progressive keratoconus: Preliminary results. J Refract Surg.

[10] Vinciguerra P, Albe E, Trazza S, Rosetta P, Vinciguerra R, Seiler T (2009). Refractive, topographic, tomographic, and aberrometric analysis of keratoconic eyes undergoing corneal cross-linking. Ophthalmology.

[11] Hafezi F, Wiltfang R, Kanellopoulos J, Seiler T (2007). Corneal collagen crosslinking with riboflavin/UVA for the treatment of induced keratectasia after laser *in situ* keratomileusis. J Cat Refract Surg.

[12] Vinciguerra P, Camesasca FI, Albe E, Trazza S (2009). Corneal collagen cross-linking for ectasia after excimer laser refractive surgery: 1-year results. J Refract Surg.

[13] Schnitzler E, Sporl E, Seiler T (2000). Irradiation of cornea with ultraviolet light and riboflavin administration as a new treatment for erosive corneal processes, preliminary results in four patients. Klin Monatsbl Augenheilkd.

[14] Schrier A, Greebel G, Attia H, Trokel S, Smith EF (2009). *In vitro* antimicrobial efficacy of riboflavin and ultraviolet light on Staphylococcus aureus, methicillin-resistant Staphylococcus aureus, and Pseudomonas aeruginosa. J Refract Surg.

[15] Wollensak G, Aurich H, Wirbelauer C, Pham DT (2009). Potential use of riboflavin/UVA cross-linking in bullous keratopathy. Ophthalmic Res.

[16] Ehlers N, Hjortdal J, Nielsen K, Søndergaard A (2009). Riboflavin-UVA treatment in the management of edema and nonhealing ulcers of the cornea. J Refract Surg.

[17] Ehlers N, Hjortdal J (2008). Riboflavin-ultraviolet light induced crosslinking in endothelial decompensation. Acta Ophthalmol.

[18] Zamora KV, Males JJ (2009). Polymicrobial keratitis after a collagen cross-linking procedure with postoperative use of a contact lens: A case report. Cornea.

[19] Krueger RR, Ramos-Esteban JC, Kanellopoulos AJ (2008). Staged intrastromal delivery of riboflavin with UVA cross-linking in advanced bullous keratopathy: Laboratory investigation and first clinical case. J Refract Surg.

[20] Iseli HP, Thiel MA, Hafezi F, Kampmeier J, Seiler T (2008). Ultraviolet A/riboflavin corneal cross-linking for infectious keratitis associated with corneal melts. Cornea.

[21] Coskunseven E, Jankov MR, Hafezi F, Atun S, Arslan E, Arslan GD (2009). Effect of treatment sequence in combined intrastromal corneal rings and corneal collagen crosslinking for keratoconus. Cataract Refract Surg.

[22] Kamburoglu G, Ertan A (2008). Intacs implantation with sequential collagen cross-linking treatment in postoperative LASIK ectasia. J Refract Surg.

[23] Chan CC, Sharma M, Wachler BS (2007). Effect of implantation of inferior-segment Intacs with and without C3-R on keratoconus. J Cataract Refract Surg.

[24] Kanellopoulos AJ, Binder PS (2007). Collagen cross-linking (CCL) with sequential topography-guided PRK: A temporizing alternative for keratoconus to penetrating keratoplasty. Cornea.

[25] Kymionis GD, Kontadakis GA, Kounis GA, Portaliou DM, Karavitaki AE, Magarakis M (2009). Simultaneous topography-guided PRK followed by corneal collagen cross-linking for keratoconus. J Refract Surg.

[26] Wollensak G, Spoerl E, Reber F, Seiler T (2004). Keratocyte cytotoxicity of riboflavin/UVAtreatment *in vitro*. Eye.

[27] Wollensak G, Spoerl E, Wilsch M, Seiler T (2004). Keratocyte apoptosis after corneal collagen cross-linking using Riboflavin/UVA treatment. Cornea.

[28] Wollensak G, Spoerl E, Wilsch M, Seiler T (2003). Endothelial cell damage after riboflavin-ultraviolet: A treatment in the rabbit. J Cataract Refract Surg.

[29] Seiler T, Hafezi F (2006). Corneal cross-linking-induced stromal demarcation line. Cornea.

[30] Mazzotta C, Balestrazzi A, Traversi C, Baiocchi S, Caporossi T, Tommasi C (2007). Treatment of progressive keratoconus by riboflavin-UVA-induced cross-linking of corneal collagen: Ultrastructural analysis by Heidelberg Retinal Tomograph II *in vivo* confocal microscopy in humans. Cornea.

[31] Kymionis GD, Kounis GA, Portaliou DM, Grentzelos MA, Karavitaki AE, Coskunseven E (2009). Intraoperative pachymetric measurements during corneal collagen cross-linking with riboflavin and ultraviolet A irradiation. Ophthalmology.

[32] Samaras K, O'brart DP, Doutch J, Hayes S, Marshall J, Meek KM (2009). Effect of epithelial retention and removal on riboflavin absorption in porcine corneas. J Refract Surg.

[33] Rabinowitz YS (2007). Intacs for keratoconus. Curr Opin Ophthalmol.

[34] Miranda D, Sartori M, Francesconi C, Allemann N, Ferrara P, Campos M (2003). Ferrara intrastromal corneal ring segments for severe keratoconus. J Refract Surg.

[35] Kymionis GD, Kontadakis GA, Naoumidi TL, Kazakos DC, Giapitzakis I, Pallikaris IG (2009). Conductive keratoplasty followed by collagen cross-linking with Riboflavin-UV-A in patients with keratoconus. Cornea.

[36] Raiskup F, Hoyer A, Spoerl E (2009). Permanent corneal haze after riboflavin-UVA-induced cross-linking in keratoconus. J Refract Surg.

[37] Mazzotta C, Balestrazzi A, Baiocchi S, Traversi C, Caporossi A (2007). Stromal haze after combined riboflavin-UVA corneal collagen cross-linking in keratoconus: *In vivo* confocal microscopic evaluation. Clin Exp Ophthalmol.

[38] Kymionis GD, Bouzoukis DI, Diakonis VF, Portaliou DM, Pallikaris AI, Yoo SH (2007). Diffuse lamellar keratitis after corneal crosslinking in a patient with post-laser *in situ* keratomileusis corneal ectasia. J Cataract Refract Surg.

[39] Kymionis GD, Portaliou DM, Bouzoukis DI, Suh LH, Pallikaris AI, Markomanolakis M (2007). Herpetic keratitis with iritis after corneal crosslinking with riboflavin and ultraviolet A for keratoconus. J Cataract Refract Surg.

[40] Koppen C, Vryghem JC, Gobin L, Tassignon MJ (2009). Keratitis and corneal scarring after UVA/riboflavin cross-linking for keratoconus. J Refract Surg.

